# The Global Campaign to Eliminate Leprosy

**DOI:** 10.1371/journal.pmed.0020341

**Published:** 2005-12-27

**Authors:** Andrea Rinaldi

## Abstract

While effective drug treatments have reduced the global disease burden, there remain important challenges to fighting and controlling the disease.

Although leprosy is no longer a health problem in developed countries, it continues to affect millions of people in large parts of Asia, Africa, and Latin America. Effective chemotherapeutic treatments are available that have reduced the global disease burden dramatically, but there remain important challenges to fighting and controlling the disease.

## Clinical Features

**Figure 1:**
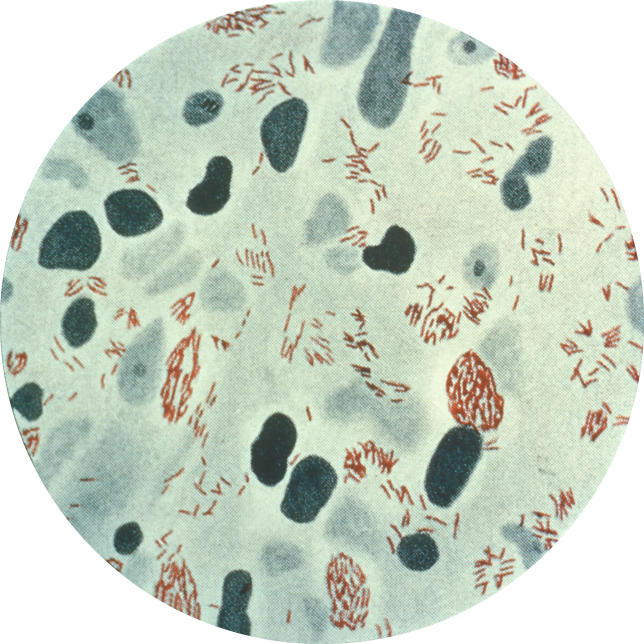
Photomicrograph of Mycobacterium leprae Taken from a Leprosy Skin Lesion (Photo: Centers for Disease Control and Prevention)

Leprosy is a chronic infection of the skin and peripheral nerves, caused by the obligate intracellular bacterium Mycobacterium leprae, the “Hansen's Bacillus” (Figure 1) [1]. Mainly transmitted by the aerosol spread of nasal secretions, the first symptoms may appear after an incubation period (usually five to ten years) following infection, and the onset is intermittent and gradual.

Skin lesions and nerve damage are the main clinical features of the disease. Blindness may develop, resulting either from destruction of peripheral nerves within ocular tissues or from direct bacillary corneal invasion. Some patients—particularly those with lepromatous leprosy (see below)—may be affected by bacillary infiltration into the mucosa of the upper respiratory tract, bones, and testes [2]. Diagnosis of leprosy is mostly clinical and symptomatic, based on the presence of a few cardinal signs: hypopigmented or reddish-copper patches with definite sensory loss, with or without thickened nerves, and positive skin smears [3].

The social consequences for those affected with leprosy and for their families can be devastating.

The disease occurs in a wide spectrum of forms, which stem from the varying host immune response to the pathogen. Individuals with tuberculoid leprosy display a strong cell-based immune response that controls bacterial proliferation and lesions, whereas patients with lepromatous leprosy lack specific cellular immunity, ending up with high mycobacterial loads and severe clinical manifestations. Most patients have borderline forms. After the age of puberty, leprosy has a male to female ratio of 1.5–2.0 to 1. Britton and Lockwood have pointed out that this male preponderance is real—it is not related to underdiagnosis in women, although in some countries it is accentuated by delayed presentation by female patients, which results in higher rates of deformity [2].

**Figure 2:**
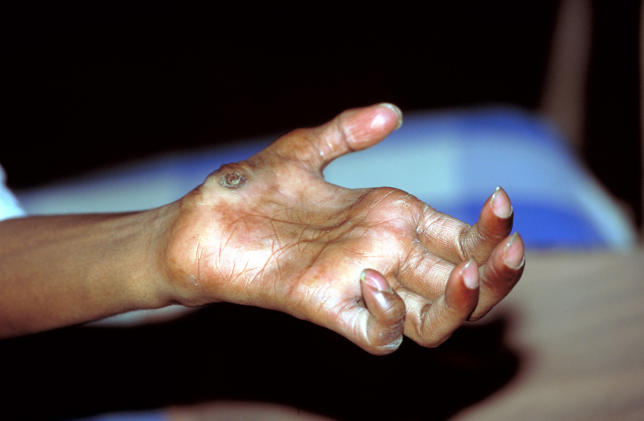
Typical Deformity in a Patient with Leprosy (Photo: copyright WHO/P.Virot. This photo may not be reproduced for commercial purposes; see http://www.who.int/about/copyright/en/index.html)

The impairment of nerve function is due both to involvement of nerves by the primary infection, and to the acute immunological phenomena known as reversal reactions or type-1 leprosy reactions. These reactions occur in a third of patients with borderline forms of disease, are caused by spontaneous increases in T-cell reactivity to mycobacterial antigens, and are associated with the infiltration of interferon γ and TNFα-secreting CD4-positive lymphocytes in skin lesions and nerves, resulting in oedema and painful inflammation [2]. Sensory loss makes affected patients prone to inadvertent injury, leading to severe disabilities and visible deformities (Figure 2).

## Burden of Disease and Disability

Given the lifelong effects of nerve damage and its consequent disabilities—which often affect very young people—and the pressure this burden of disease and disability poses on fragile medical systems, the prevention, detection, and management of nerve function impairment are pivotal to all leprosy-control programmes [2].

Foot ulcers alone, for example, which are common in anaesthetic feet, can pose a huge burden on medical services. The social consequences for those affected with leprosy and for their families can be devastating. Stigma, community rejection, loss of employment, and sometimes forced isolation are still prevalent in both endemic and non-endemic countries [4].

## The Biology of M. Leprae

One of the oldest recorded diseases [5], leprosy was also the first human pathogenic condition of bacterial origin for which the causative agent was identified. Despite these historical records, our knowledge of the biology of M. leprae has lagged behind, in good part because it cannot be grown in culture.

The full sequencing of the M. leprae genome, completed in 2001, has created possibilities for the development of new diagnostic tests and treatments for leprosy [6]. Analysis of the M. leprae genome has revealed that it contains fewer than half the functional genes of its closest relative, the tubercule bacillus M. tuberculosis [6,7]. This “minimal gene set”, the result of extensive gene deletion and decay that have eliminated many key metabolic pathways, renders the leprosy mycobacterium extremely slow in replicating and forces it to an intracellular existence.

A massive international effort was launched to eradicate leprosy worldwide.

In some areas such as the Middle East and Europe, leprosy declined after the late medieval period. One theory for the decline is that it was related to the increasing prevalence of tuberculosis—cross-immunity may have protected patients with tuberculosis from developing leprosy, or the compromised immunological status of patients with leprosy may have rendered them more susceptible to underlying latent tuberculosis infection, which resulted in increased mortality [8].

## The Global Elimination Campaign

Leprosy now occurs mainly in resource-poor countries in tropical and warm temperate regions. Contrary to a widely believed myth, nowadays leprosy is a fully curable disease. A multidrug therapy (MDT) based on the combination of the antibiotics dapsone, rifampicin, and clofazimine was introduced in 1982 after dapsone-resistant strains appeared and spread. MDT proved highly efficacious in killing the bacteria without inducing resistance, although the optimal length of treatment and associated relapse rates are still controversial [2].

With such a powerful weapon at hand, a massive international effort was launched to eradicate leprosy worldwide. In 1991, the World Health Assembly adopted the target of “elimination of leprosy as a public health problem by the year 2000”. Elimination was defined as a reduction in the prevalence of patients with leprosy receiving antimicrobial therapy at a given time to less than 1 per 10,000 population. It was expected that by reducing the prevalence to this level, the transmission of M. leprae would be interrupted, leading to the gradual extinction of the disease. Since its introduction, some 13 to 14 million people have been cured with MDT (made available free of cost by the Sasakawa Foundation and then by Novartis), and full control of the disease (as assessed by prevalence rate) has been officially achieved in 112 of the 122 countries where leprosy was endemic in 1985.

## The Final Push

The World Health Organization (WHO) dubbed the ambitious project “the final push to eliminate leprosy”. The strategy behind the slogan involves expanding MDT services to all health facilities and making leprosy diagnosis available, training health workers to diagnose and treat leprosy, promoting leprosy awareness and encouraging people to seek and continue treatment [9]. However, despite the impressive results obtained so far by the elimination campaign, this is still a work in progress.

According to the last WHO report (for 2003), ten countries in Africa, Asia, and Latin America still show prevalence rates above the selected threshold [10]. Topping this short list is a group of six endemic countries that together account for 83% of the leprosy cases registered worldwide: India, Brazil, Madagascar, Mozambique, Nepal, and Tanzania. According to WHO, in 2004 the number of patients with leprosy worldwide was 457,792 [11].

“We shall not be able to eliminate leprosy until we have a better understanding of its natural reservoir.”

Worryingly, whereas prevalence figures have fallen steadily in the last two decades, the annual rate of new cases did not follow a comparable trend—this rate has remained essentially unchanged over the past ten years. Indeed, the number of new cases detected during 1994 was 560,646, increasing to 804,357 in 1998, then falling again to 513,798 in 2003 [10,11]. On the basis of available information, WHO considers the “global target of leprosy elimination” as reached, and has shifted the strategy to the national level, for which elimination has been rescheduled for the end of 2005 [10]. In its plans, WHO estimates that eight out of the remaining ten countries will reach the new target, while India and Brazil will probably need additional time.

The key constraints to eliminating leprosy in those countries that lag behind the elimination campaign vary greatly from country to country. In some leprosy-endemic countries (such as Madagascar, Mozambique, Nepal, and Tanzania), access to many health facilities is extremely poor because of difficult terrain, displacement of populations in remote areas, or for security reasons [10]. In other countries, such as Brazil, important problems arise from the very centralised structure of the leprosy programme, and from its poor integration with general health services [10]. To deal with these very different scenarios, the strategies identified by WHO vary accordingly, proposing in some cases the complete restructuring of the national leprosy programmes [10].

## A New Strategy for an Uncertain Future

So what will happen after 2005? Leprologists and people involved in disease control fear that once leprosy is declared “eliminated as a public health problem”, the future of anti-leprosy services and of leprosy workers and researchers will be at high risk [12,13]. Elimination is not eradication, many warn, and it must be clear to everyone that leprosy will continue to exist even in areas where the “elimination goal” has officially been reached. The term elimination itself makes people think the problem is over, say critics of the WHO policy, which can have detrimental effects on the future commitment of governments to sustain control activities, making it at the same time difficult for leprosy NGOs and scientists to raise funds for field and lab work.

Others believe that the concept of elimination itself, and the choice of prevalence as an indicator to measure the progress of the WHO-orchestrated campaign, are scientifically devoid of significance—as is the 2005 deadline. “As a matter of fact, the wrong indicator has been selected to reflect the progress toward elimination of leprosy,” says Piet Feenstra at the Royal Tropical Institute of Amsterdam (Amsterdam, The Netherlands), remarking that new-case detection and the proportion of children among new cases would serve much better to monitor the real disease status.

The “elimination” strategy must be swiftly converted to a “post-elimination” strategy.

The International Leprosy Association's Technical Forum has also noted that the expectation that reduction of prevalence to very low levels would lead to a reduction of the incidence within a few years was overoptimistic, as there was little evidence to support this hypothesis [14]. Since patients are only registered while they are on medication, prevalence figures by WHO standards vary depending on how long treatment lasts. “The decrease of prevalence is attributable primarily to the cleaning of the registers (discharge of cured or defaulting patients), to shortening the duration of treatment and, in some countries, to improved diagnostic accuracy, and is not a consequence of reduction of the transmission of Mycobacterium leprae,” Feenstra says.

“I believe there is probably a lot more leprosy in the world than the World Health Organization currently accepts,” agrees Helen Donoghue, a leprosy researcher at the Windeyer Institute of Medical Sciences (London, United Kingdom). The political implications of the “elimination goal”, and the way it was enforced by WHO, have also been questioned. “Over the last years, the elimination target has more and more become a political target [rather] than an epidemiological or program quality target,” says Feenstra. “For many, the indicator—the prevalence of patients registered for treatment—has become the goal in itself, and the actual goal—reduction of the leprosy transmission and incidence—has practically got out of sight”. Furthermore, the fixing of numerical targets may put excessive pressure on national leprosy programme managers, discouraging them from actively working to detect new cases, which in turn could jeopardise the country's elimination status.

## Cracks in the Coalition

Another cause for serious concern is that the coalition that stands against leprosy is not as solid as it should be. In 1999, the Global Alliance for the Elimination of Leprosy (GAEL) was formed to inject new energy into the elimination campaign, bringing together WHO, the governments of the major endemic countries, the Japanese Nippon Foundation, the Novartis Foundation for Sustainable Development, the Danish Development Cooperation Agency (Danida), and the International Federation of Anti-Leprosy Associations (ILEP). Quite soon, major contrasts emerged between some of the GAEL partners, namely between WHO and ILEP, who always remained critical of the “elimination”-focussed strategy. The clash was so strong that ILEP was expelled from the alliance at the end of 2001.

Later on, probably in response to the increasing pressure to achieve leprosy control, WHO invited an independent team of experts led by Richard Skolnik, former Director of the Center for Global Health at George Washington University (Washington, District of Columbia, United States), to evaluate the GAEL. The evaluation report, published in 2003, recommends that WHO should take leprosy activities beyond 2005, dropping the “elimination” goal in favour of “an explicitly broad-based approach to the control of leprosy, the avoidance of nerve damage, and the rehabilitation of those in need” [15]. The team also explicitly called for the reconstitution of a refined alliance, where “collaborators will have to work more openly, collegially, and inclusively” [15].

There are signs that this new alliance is emerging. “The process of dialogue and collaboration with WHO headquarters in Geneva has already been reestablished and is improving constantly,” says Sunil Deepak, president of ILEP and medical director of the Italian leprosy NGO Associazione Italiana Amici di Raoul Follerau (AIFO). Deepak adds, “We are very optimistic about further strengthening of this collaboration”. Feenstra confirms that stakeholders are exploring new ways of dialogue. “WHO is currently, in collaboration with its partners, ILEP, The Nippon Foundation and Novartis, developing a new strategy for the period 2006–2010 for sustaining quality leprosy control activities,” he says (also see [16]).

## A “Post-Elimination” Strategy

John Porter from the London School of Hygiene and Tropical Medicine (London, United Kingdom) recently argued that in order to make sure “the disease does not go underground”, the “elimination” strategy must be swiftly converted to a “post-elimination” strategy [12]. As recommended by many leprosy experts, this post-elimination strategy should focus on integrating leprosy control activities into primary health care services, assuring early case detection, adequate chemotherapy, prevention of disability for all patients with nerve damage, and physical rehabilitation of those already disabled [12,17,18].

Work to dispel the stigma of leprosy and to introduce patients back into their communities must also be strengthened, experts note, in order to end social discrimination toward people with leprosy [18]. Leprosy remains a disease of the poor, although the exact social factors that put people at risk have not been identified [19]. To break this link between leprosy and poverty, “leprosy should…now be included in the portfolio of diseases associated with poverty, and leprosy work incorporated into poverty reduction programmes,” points out Diana Lockwood of the London School of Hygiene and Tropical Medicine [20].

Lockwood and Suneetha have also suggested that the routine use of vaccination could benefit the outcome of WHO's anti-leprosy strategy [18]. Although the development of a specific and highly effective vaccine against leprosy is not yet a reality, the tuberculosis vaccine bacillus Calmette-Guérin, made with live attenuated Mycobacterium bovis with the eventual addition of heat-killed M. leprae, has been proven to offer some immunity to leprosy. Its reported efficacy ranges from 34% to 80% in different countries [2]. Despite this variable efficacy, bacillus Calmette-Guérin vaccination is already widely used in leprosy-endemic countries—but who should be vaccinated, when, and how often in order to achieve maximal protection among the population are all a matter for debate [21]. Another promising intervention for leprosy prevention comes from a recent study, conducted on five Indonesian islands, that found that giving people who are in close contact with patients with leprosy a short course of rifampicin can reduce their risk of developing the disease [22].

Finally, important research issues remain to be addressed. They include developing improved diagnostic tests and better ways to monitor and treat nerve damage, and understanding why MDT has not interrupted transmission [18]. “We shall not be able to eliminate leprosy until we have a better understanding of its natural reservoir,” says Donoghue. “There are several interesting reports that indicate that there may be an environmental reservoir for M. leprae—perhaps even in the soil in endemic regions.” Healthy human carriers also do exist, says Donoghue, who points out that by using sensitive molecular methods it is possible to detect the DNA from M. leprae in people who showed non-specific skin lesions and who had not been thought to have leprosy. Even after a ten-year MDT programme, more than 5% of healthy individuals in a leprosy-endemic area were positive for M. leprae DNA in their nasal passages, a recent study found, suggesting a high level of environmental contamination [23]. “Before anyone can talk about eliminating the disease we have to understand where the organisms are found, and the circumstances that result in an active infection,” Donoghue says.

## Conclusion

It is widely believed that the leprosy elimination campaign has been a positive one. A comparably large consensus must now come together around the post-2005 leprosy agenda, to make sure that we do not lose the gains achieved to date or miss this unique opportunity to reach complete control of leprosy. Political, medical, or scientific disputes may have previously impeded unity among WHO and other stakeholders, but a reinforced partnership is now needed to continue the struggle to control and eradicate the disease.

## Abbreviations

GAEL: Global Alliance for the Elimination of Leprosy
ILEP: International Federation of Anti-Leprosy Associations
MDT: multidrug therapy
WHO: World Health Organization

## References

[pmed-0020341-b1] Ustianowski AP, Lockwood DNJ (2003). Leprosy: Current diagnostic and treatment approaches. Curr Opin Infect Dis.

[pmed-0020341-b2] Britton WJ, Lockwood DNJ (2004). Leprosy. Lancet.

[pmed-0020341-b3] World Health Organization (2005). Leprosy. http://www.who.int/lep/disease/disease.htm.

[pmed-0020341-b4] Withington SG, Joha S, Baird D, Brink M, Brink J (2003). Assessing socio-economic factors in relation to stigmatization, impairment status, and selection for socio-economic rehabilitation: A 1-year cohort of new leprosy cases in north Bangladesh. Lepr Rev.

[pmed-0020341-b5] Ostrer BS (2002). Leprosy: Medical views of Leviticus Rabba. Early Sci Med.

[pmed-0020341-b6] Cole ST, Eiglmeier K, Parkhill J, James KD, Thomson NR (2001). Massive gene decay in the leprosy bacillus. Nature.

[pmed-0020341-b7] Vissa VD, Brennan PJ (2001). The genome of Mycobacterium leprae: A minimal mycobacterial gene set. Genome Biol 2. http://genomebiology.com/2001/2/8/reviews/1023.

[pmed-0020341-b8] Donoghue HD, Marcsik A, Matheson C, Vernon K, Nuorala E (2005). Co-infection of Mycobacterium tuberculosis and Mycobacterium leprae in human archaeological samples: A possible explanation for the historical decline of leprosy. Proc R Soc Lond B Biol Sci.

[pmed-0020341-b9] World Health Organization (2000). Guide to eliminate leprosy as a public health problem. http://www.who.int/lep/disease/Eliminate_Leprosy_V8.pdf.

[pmed-0020341-b10] World Health Organization (2004). Leprosy elimination project. Status report 2003.

[pmed-0020341-b11] World Health Organization (2005). Global leprosy situation 2004. http://www.who.int/lep/stat2002/global02.htm.

[pmed-0020341-b12] Porter JDH (2004). Supporting ‘the individual’ with leprosy: The need for a ‘post-elimination strategy’. Lepr Rev.

[pmed-0020341-b13] Faber WR, Klatser PR (2003). Elimination of leprosy and its consequences for research. Adv Exp Med Biol.

[pmed-0020341-b14] International Leprosy Association (2002). Report of the International Leprosy Association's technical forum. Lepr Rev.

[pmed-0020341-b15] Skolnik R, Agueh F, Justice J, Lechat M (2003). Independent evaluation of the Global Alliance for the Elimination of Leprosy. http://gujhealth.gov.in/health_programmes/pdf/leprosy/GAEL%20Evaluation%20Report.pdf.

[pmed-0020341-b16] World Health Organization (2005). Global strategy for further reducing the leprosy burden and sustaining leprosy control activities. http://www.who.int/lep/Reports/GlobalStrategy-PDF-verison.pdf.

[pmed-0020341-b17] Visschedijk J, Engelhard A, Lever P, Grossi MA, Feenstra P (2003). Leprosy control strategies and the integration of health services: An international perspective. Cad Saude Publica.

[pmed-0020341-b18] Lockwood DNJ, Suneetha S (2005). Leprosy: Too complex a disease for a simple elimination paradigm. Bull World Health Organ.

[pmed-0020341-b19] Kerr-Pontes LRS, Montenegro ACD, Barreto ML, Werneck GL, Feldmeier H (2004). Inequality and leprosy in northeast Brazil: An ecological study. Int J Epidemiol.

[pmed-0020341-b20] Lockwood DNJ (2004). Leprosy and poverty. Int J Epidemiol.

[pmed-0020341-b21] Smith WCS (2004). What is the best way to use BCG to protect against leprosy: When, for whom, and how often?. Int J Lepr Other Mycobact Dis.

[pmed-0020341-b22] Bakker MI, Hatta M, Kwenang A, Van Benthem BH, Van Beers SM (2005). Prevention of leprosy using rifampicin as chemoprophylaxis. Am J Trop Med Hyg.

[pmed-0020341-b23] Beyene D, Aseffa A, Harboe M, Kidane D, Macdonald M (2003). Nasal carriage of Mycobacterium leprae DNA in healthy individuals in Lega Robi village, Ethiopia. Epidemiol Infect.

